# Excitonic structure and charge separation in the heliobacterial reaction center probed by multispectral multidimensional spectroscopy

**DOI:** 10.1038/s41467-021-23060-9

**Published:** 2021-05-14

**Authors:** Yin Song, Riley Sechrist, Hoang H. Nguyen, William Johnson, Darius Abramavicius, Kevin E. Redding, Jennifer P. Ogilvie

**Affiliations:** 1 Department of Physics, University of Michigan, Ann Arbor, MI USA; 2 School of Molecular Sciences, Arizona State University, Tempe, AZ USA; 3 Institute of Chemical Physics, Faculty of Physics, Vilnius University, Sauletekio, Vilnius, Lithuania; 4 Center for Bioenergy and Photosynthesis, Arizona State University, Tempe, AZ USA

**Keywords:** Kinetics, Energy transfer, Excited states, Biological physics

## Abstract

Photochemical reaction centers are the engines that drive photosynthesis. The reaction center from heliobacteria (HbRC) has been proposed to most closely resemble the common ancestor of photosynthetic reaction centers, motivating a detailed understanding of its structure-function relationship. The recent elucidation of the HbRC crystal structure motivates advanced spectroscopic studies of its excitonic structure and charge separation mechanism. We perform multispectral two-dimensional electronic spectroscopy of the HbRC and corresponding numerical simulations, resolving the electronic structure and testing and refining recent excitonic models. Through extensive examination of the kinetic data by lifetime density analysis and global target analysis, we reveal that charge separation proceeds via a single pathway in which the distinct A_0_ chlorophyll *a* pigment is the primary electron acceptor. In addition, we find strong delocalization of the charge separation intermediate. Our findings have general implications for the understanding of photosynthetic charge separation mechanisms, and how they might be tuned to achieve different functional goals.

## Abstract (web summary)

The primary energy conversion step in photosynthesis, charge separation, takes place in the reaction center. Here the authors investigate the heliobacterial reaction center using multispectral two-dimensional electronic spectroscopy, identifying the primary electron acceptor and revealing the charge separation mechanism.

## Introduction

Photosynthesis drives life on Earth by converting solar energy into chemical energy^1,2^. In photosynthesis, light is absorbed by the antenna pigments, which transfer their excitation energy to the photochemical reaction center (RC)^2,3^. The primary energy conversion step, charge separation (CS), takes place in the RC^2^, driving the subsequent chemical reactions for synthesizing high-energy chemical compounds. All RCs are believed to have evolved from a common homodimeric ancestor RC, in which a dimer of a single polypeptide binds the redox-active cofactors^4,5^. Until recently, the crystal structures of the existing homodimeric RCs were unknown^6^, hindering mechanistic understanding of these systems. Recently, the crystal structure of the heliobacterial RC (HbRC) from *Heliobacterium modesticaldum*, was characterized by Gisriel and coworkers^6^. The HbRC has been proposed to be the RC most similar to the common ancestor of all photosynthetic RCs^4,5^. Furthermore, it is the simplest known RC and structural analog to the photosystem I RC^7^ (PSI RC), and possesses three chemically distinct pigments.

**Fig. 1:**
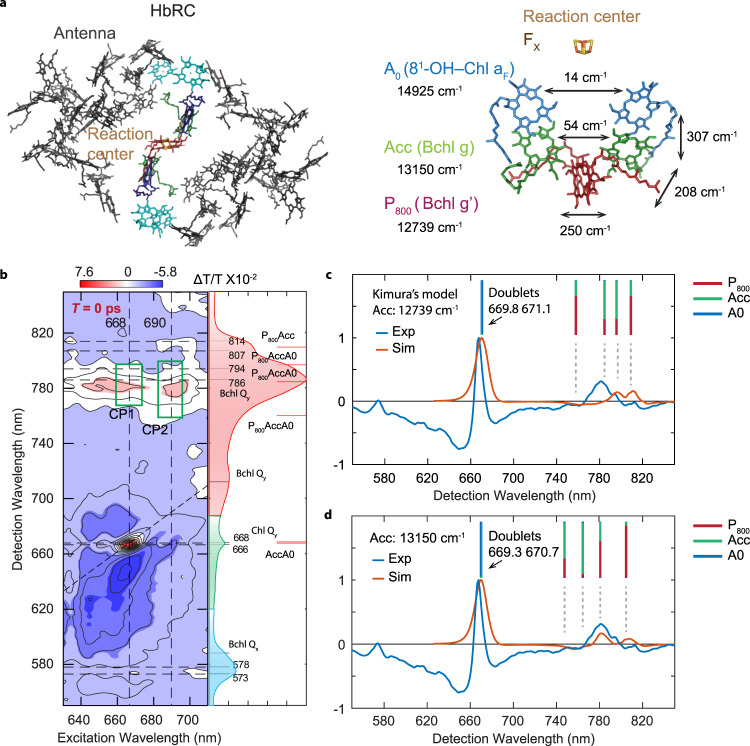
Experimental and simulated 2DES absorptive spectra at *T* = 0 ps reveal the excitonic structure of the core RC at 77 K. **a** The pigment arrangement in the full HbRC (left) and in the core RC (electron transfer domain). The same color scheme is used in the full HbRC and the core RC. The arrows and the numbers on top show the electronic coupling strengths between two pigments. **b** The experimental 2DES absorptive spectrum at *T* = 0 ps. Green boxes from left to right indicate two cross-peaks positions (i.e., CP1 and CP2) discussed in the main text. Alongside the 2D absorptive spectra are the experimental absorption spectrum and the simulated stick spectrum (red lines) from Kimura et al^8^. The experimental absorption spectrum is divided into three color-coded regions based on their dominant contributions (i.e., red—the Bchl Q_y_ band; green—the Chl Q_y_ band; blue—the Bchl Q_x_ band). The dashed lines in the 2DES spectrum and the gray lines in the absorption spectrum indicate the various peak positions discussed in the main text. The contour levels are −1:0.1:1 of the maximum amplitude. **c** The experimental and simulated slice spectra under excitation of A_0_ (slice position shown as vertical dashed line at excitation wavelength = 668 nm) at *T* = 0 ps extracted from the 2DES absorptive spectra. The site energies of BChl g and BChl g′ are set to be equal according to Kimura’s excitonic model. **d** The experimental and simulated slice spectra under excitation at 668 nm at *T* = 0 ps extracted from the 2DES absorptive spectra. Modifying the site energy of accessory BChl g to be 13150 cm^−1^ yields considerably better agreement with the experimental data. The presence of distinct BChl Q_y_ peaks upon excitation at 668 nm reveals excitonic coupling between A_0_ and the BChl pigments Acc and P_800_. The stick bars reveal the participation ratios of the RC pigments in each excitonic state as given in the respective exciton models.

The HbRC binds 54 BChl g, four BChl g′ (the 13^2^-sterochemical isomer), two 8^1^-hydroxychlorophyll *a*_*F*_ (8^1^-OH Chl a), two carotenoids (4,4′-diaponeurosporene), and one [4Fe-4S] cluster^6^. The majority of the BChl g pigments serve as an antenna to direct excitation to the homodimeric core, which serves as the electron transfer domain, depicted in Fig. 1. Throughout this paper we adopt the language commonly used in discussing RCs, where we reserve the term RC for the electron transfer domain, and the other pigments are collectively referred to as the “antenna”. The homodimeric RC consists of two symmetric branches, each containing a BChl g (Acc) and Chl a (A_0_), arranged about the central symmetry axis running between the BChl g′ dimer (P_800_) and [4Fe-4S] cluster (F_X_)^6^. A structure-based excitonic model of the full HbRC was recently proposed by Kimura and Itoh^8^. They reported that the HbRC pigments form 58 excitonic states with 0–0 transition energies spanning 771 to 812 nm and two excitonic states with dominant absorption peaks centered at 667 nm. Until now, this model has not been tested by advanced experimental techniques.

In comparison to other RCs, the HbRC combines Chl a and BChl g, which possess distinct spectroscopic properties and redox potentials, making it a particularly appealing system for spectroscopic studies of its primary CS processes and structure-function relationship. Energy transfer and CS in the HbRC have been studied by several groups^9–19^, with the early work reviewed by Neerken et al^15^. In light of previous proposals of the CS mechanism and the new structure, Gisriel et al. proposed two possible charge-separation mechanisms: one in which Acc acts as the initial electron acceptor, and another in which Acc acts as the initial electron donor^6^, with CS occurring with equal probability along either branch of the homodimer^6^.

Two-dimensional electronic spectroscopy (2DES) has proven to be a powerful tool to study photoexcited dynamics in a variety of systems including light-harvesting complexes^20–22^, photosynthetic RCs^23,24^, and solar cells^25,26^. In 2DES, a pair of pump pulses prepare an excited state population, the evolution of which is monitored by a probe pulse as a function of “waiting time” *T*. The 2DES spectrum at *T* = *0* is a frequency-frequency map that directly highlights correlations between photoexcited and photodetected states via cross-peaks induced by excitonic interactions. The *T*-dependence of the 2DES spectra reveals the evolution of excited state populations, enabling the testing of kinetic models and the identification of spectroscopic fingerprints of reaction intermediates. Thus, 2DES is closely related to transient absorption spectroscopy, but provides simultaneous time and frequency resolution by opening an additional dimension, resolving the same third order material response of the system with respect to the excitation frequency. Here we have applied 2DES to the HbRC, revealing excitonic correlations between Acc, A_0_, and P_800_ and refining the proposed excitonic structure. We find that CS in the HbRC proceeds via a two-step process in which A_0_ acts as the primary electron acceptor. Moreover, our results suggest that the hole is initially delocalized over Acc and P_800_ immediately after the primary CS event, but later becomes localized at P_800_^+^.

## Results

To probe the HbRC excitonic structure and accentuate CS processes originating from RC excitation, we excited the ~590–720 nm region, spanning the Q_y_ A_0_ Chl and vibronic shoulder of Q_y_ BChl g transitions. The multispectral probes in our recently developed 2DES instrument^27^ access both Q_x_ and Q_y_ of Chls and BChls, providing dynamical information about energy transfer and CS processes. We also performed complementary 2DES studies with excitation of the BChl g Q_y_ region.

### Excitonic structure of the HbRC

Figure 1b displays 2DES absorptive spectra of the HbRC at 77 K, in which the sample was treated with sodium dithionite to reduce the F_X_ cluster. This blocks forward electron transfer from A_0_ to F_x_, resulting in charge recombination of the P_800_^+^A_0_^−^ state in ~15 ns^10^. At *T* = 0 ps, the 2D absorptive spectrum, which combines rephasing and nonrephasing contributions, provides insight into the HbRC’s excitonic structure and tests the current structure-based excitonic model^8^. We note that the term “exciton” is often reserved for strongly-mixed, delocalized states. Here we use the term more broadly to describe the eigenstates of the singly-excited manifold of the coupled HbRC system. The diagonal peak at 668 nm is consistent with photoexcitation of the highest energy RC excitons assigned to two states, primarily involving A_0_ with some participation from Acc^8^. The presence of cross peaks in the region λ_ex_ = 668 nm and λ_det_ = 770–800 nm (denoted CP1) is key to understanding the pigment composition of the excitonic states in the crowded BChl Q_y_ region. The model of Kimura and Itoh^8^ predicts roughly three states in the 760–800 nm region that involve participation from A_0_ and could therefore produce CP1 in the *T* = 0 2D spectrum. These states, as well as the other RC excitons from the Kimura model, are indicated by solid red lines in the linear absorption spectrum shown in Fig. 1b (right panel). To test the Kimura exciton model, we simulated the 2DES absorptive spectrum in the CP1 region. The simulations employ the harmonic bath model within the modified Redfield approach to account for the spectral lineshape. Optical response functions are obtained within the adiabatic limit during electronic coherence periods: diagonal system–bath fluctuations in the eigenstate representation are included using the second-order cumulant expansion, and the corresponding off-diagonal fluctuations are included within the Markovian second-order perturbation theory^28,29^. Static site energy fluctuations are included by ensemble averaging over Gaussian site energy disorder. All simulation parameters are provided in the Supplementary Note 6. The resulting “slice spectrum”, which plots the detection frequency dependence at a specific excitation wavelength, is shown in Fig. 1c at *T* = 0 ps for excitation of A_0_. The slice spectrum reveals excitonic correlations that can be obscured in transient absorption measurements of systems that exhibit ultrafast excited state evolution. The two cross peaks in the BChl Q_y_ band provide firm evidence for excitonic correlation, but are red-shifted relative to the experimental data, indicating that refinement of the excitonic parameters is needed to capture the tuning of pigment site energies by the protein environment, which is not included in the Kimura exciton model^8^. We find better agreement between experiment and theory by shifting the site energy of Acc from 12,739 cm^−1^ (785 nm) to 13,150 cm^−1^ (760 nm (see Fig. 1d). We also note that due to strict C_2_ symmetry of the HbRC, excitonic states are delocalized between the symmetric pigments, and, hence, some are suppressed, while others become superradiant. Although some of the couplings for pigments across the branches of the RC are relatively weak (such as 14 cm^−1^ between A_0_ pigments), each of the RC pigments is coupled to at least one neighbor with considerable coupling strength (~200–300 cm^−1^), yielding the delocalized exciton states of the system. Site-energy disorder removes the strict C_2_ symmetry of the RC, inducing a competing localization effect. We note that some peaks involving the A_0_ pigments do not appear in the simulated 2DES, due to a combination of their low oscillator strength, and competing overlapping contributions of the ground-state bleach (GSB), stimulated emission and excited state absorption (ESA) signals. Our simulations assume a three-state model for the Q_y_ transitions and include doubly-excited states of the isolated pigments. However, they only approximately account for the ESA contributions. For example, electrochromic (Stark) shifts due to excitations of neighboring pigments are neglected in the present modeling. The broad ESA features are unlikely to affect the cross-peak positions that we use here to assess the Kimura exciton model. Further refinement of Kimura’s model with respect to 2DES and other measurements will be the subject of future work. Further simulation details are given in Supplementary Note 6 of the Supplementary Information (SI).

### Charge separation in the HbRC

**Fig. 2:**
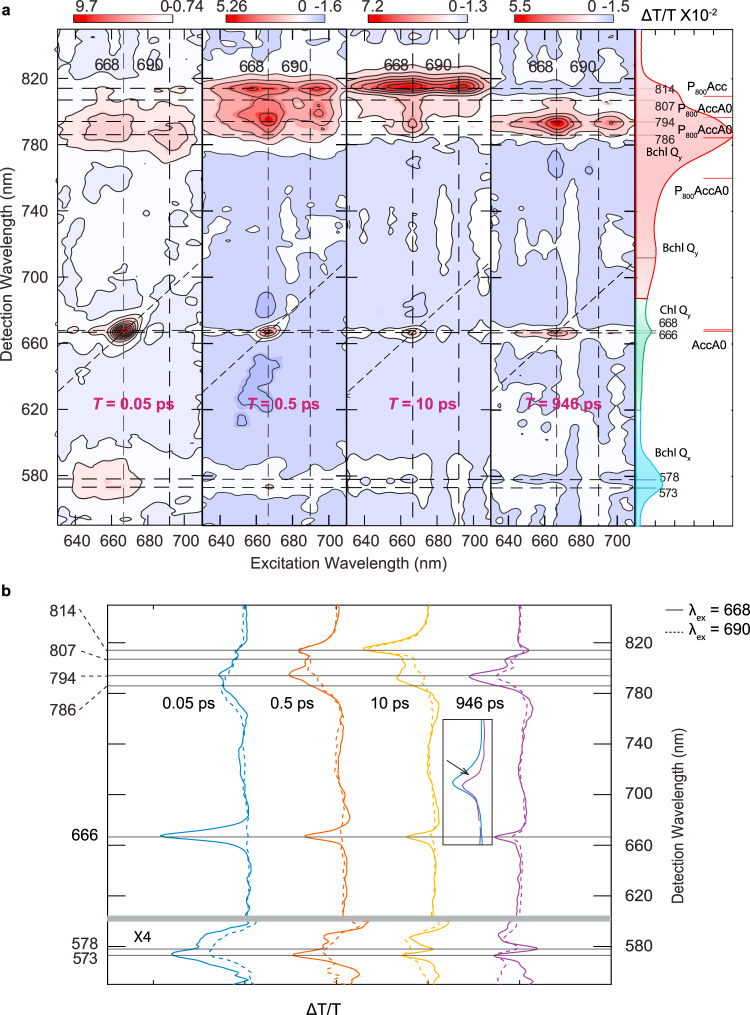
2DES probes the photoexcited dynamics in the HbRC at 77 K. **a** 2DES absorptive spectra at 0.05, 0.5, 10, and 946 ps. Alongside the 2D absorptive spectra are the experimental absorption spectrum and the simulated stick spectrum (red lines) of Kimura et al.^8^. The dashed lines in the 2DES and the gray lines in the absorption spectrum indicate the various peak positions discussed in the main text. The experimental absorption spectrum is divided into three color-coded regions based on their dominant contributions (i.e., red—the Bchl Q_y_ band; green—the Chl Q_y_ band; blue—the Bchl Q_x_ band). The contour levels are −1:0.1:1 of the maximum amplitude. **b** The slice spectra under excitation at 668 nm (dashed) and 690 nm (dashed) at various *T* extracted from the 2DES absorptive spectra. The derivative lineshapes at 573, 578 nm and the blue-shifted A_0_ peak at 666 nm (inset) are induced by the Stark effect. These features are fingerprints for the charge-separated state.

While both the RC and the antenna excitons are simultaneously excited in this experiment, the spectral signatures are separated along the excitation and detection wavelengths. This enables investigation of the CS mechanism(s) initiated by direct excitation of the RC or excitation of the antenna. The 2DES spectra in Fig. 2 show that by 0.5 ps the 668-nm diagonal peak has largely decayed, accompanied by the rise of two cross peaks at 794 nm and 814 nm. We interpret these signatures as redistribution of energy within the RC and equilibration with the antenna. The contribution of antenna excitons to these cross peaks is evident in the similarity of the cross-peak structure at an excitation wavelength of 690 nm, which corresponds to predominantly antenna excitation. At 946 ps, the 2DES absorptive spectrum has become excitation wavelength independent, suggesting that excitons have been converted to the final charge-separated state (CSS)— P_800_^+^A_0_^−^, as reported in previous studies^9,12–16,30,31^. In this 2D spectrum, a derivative lineshape near 575 nm (Q_x_ band of BChl g) shown in Fig. 2b is induced by the Stark effect due to the presence of an adjacent charge^11^, providing definitive fingerprints for CS. The CS is also evident in the evolution of the GSB peak of the 668-nm RC exciton, which shifts to λ_det_ = 666 nm. This peak shift is due to the overlap of the GSB of A_0_^−^ and the Stark shift of the neutral A_0_ in the other branch, similar to previous studies^7,11^ that observed the Stark shift associated with the formation of P_800_^+^^11^. We support our assignment with simulations of the Stark shift detailed in the Supplementary Note 7. Two other GSB peaks at λ_det_ = 794 and 807 nm decay with A_0_^−^ and can thereby be assigned to the upper and the lower excitonic peaks of P_800_^+^^10,11^.

To make full use of the rich information from 2DES, we used a combination of lifetime density analysis (LDA)^32,33^ to obtain an overview of the kinetics, followed by global-target analysis^34^ to test specific kinetic models. LDA fits the data using exponential functions with a continuous distribution of time constants. As no prior knowledge is required for this analysis, this approach is well suited to systems like the HbRC, in which multiple relaxation pathways are expected. Lifetime density maps (LDMs) that reveal the kinetics following RC and antenna excitation are shown in Supplementary Figs. 3 and 4, respectively. LDM reveals the Stark lineshapes of the BChl Q_x_ peak (Supplementary Fig. 3b1) at 1.7 and 230 ps, which identify two time-windows for CS. At 1.7 ps, the Stark lineshape of the BChl g Q_x_ peak (Supplementary Fig. 3 panels b1–b3), does not yet exhibit the clear signatures of P_800_^+^A_0_^−^ (i.e., peaks at 666, 794, and 807 nm), suggesting that an intermediate CSS exists on this timescale. The decay of the GSB of A_0_ on this timescale is consistent with the participation of A_0_ in the intermediate CSS and suggests that it is the primary electron acceptor. The LDMs for antenna excitation are consistent with this assignment, leading us to propose the following CS mechanism for the HbRC:

Upon direct excitation of the RC: (RC)* → relaxed (RC)* → intermediate CSS → P_800_^+^A_0_^−^

Upon antenna excitation: (Antenna exciton)* → relaxed (Antenna exciton) → (RC)* → intermediate CSS → P_800_^+^A_0_^−^.

**Fig. 3:**
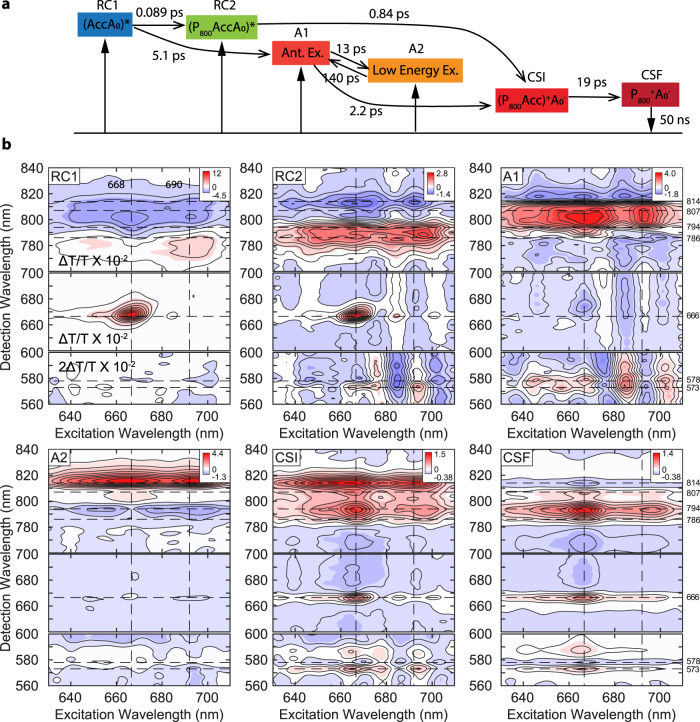
Target analysis of the HbRC reveals the kinetic map and insights into the participating states during CS. **a** The kinetic model in which CS proceeds via a two-step mechanism with A_0_ as the primary electron acceptor, independent of whether the initial excitation occurs within the RC or antenna domains. The vertical arrows indicate species that can be generated by direct photoexcitation. **b** The species-associated spectra obtained from the global-target analysis. The dashed lines in SASs indicate various peak positions as shown in Figs. 1–2 and discussed in the main text. The contour levels are −1:0.1:1 of the maximum amplitude.

With this approximate CS mechanism, we turn to global-target analysis of the 2DES data to test and refine this initial model and extract spectroscopic signatures of the intermediates. This is often used in kinetic analysis of transient absorption spectroscopy^34^ and has been applied in 2DES studies^35–39^. This approach fits the data using a trial kinetic model and produces a set of species-associated spectra (SAS) and rate constants. Guided by the LDA analysis and the revised Kimura model, we considered multiple kinetic models before arriving at the one presented in Fig. 3a. Further justification of our choice is given in Supplementary Note 4, where we discuss a subset of alternative models, including ones that incorporate additional and alternative CS pathways. The Fig. 3a model provides the best fit to the 2D data and produces SAS with spectroscopic characteristics consistent with the target model. In this model, two RC excitons and two antenna excitons are excited by our broadband pulses, after which downhill energy transfer precedes rapid CS via a single intermediate CSS. Species RC1, with GSB peaks at λ_det_ = 668 and 780 nm, and an ESA peak at λ_det_ = 810 nm, is assigned to the highest energy RC exciton (i.e., (A_0_Acc)*). This assignment is consistent with the presence of GSB peaks in the *T* = 0 2DES spectrum and is further supported by the large coupling strength of 200–300 cm^−1^ between A_0_ and Acc^8,11^ in the Kimura model. Our proposed shift of Acc’s site energy suggests a stronger degree of participation from P_800_ in this RC exciton compared to the Kimura model.

After photoexcitation, RC1 transitions to the relaxed RC exciton—RC2 with a time constant of 0.09 ps. RC2 exhibits GSB peaks at λ_det_ = 668 and 790 nm. RC2 was not observed with excitation at 690 nm (see Supplementary Fig. 6), leading us to infer that RC2 is also an RC excitonic state. Consistent with the Kimura model, we attribute RC2 to a lower energy RC exciton involving some degree of participation from all RC pigments (P_800_AccA_0_)*^8,40^. We note that RC2 exhibits a weak first-derivative feature near 575 nm that may indicate some initial charge-separated character.

RC2 transitions to CSI (CS intermediate) with a time constant of 0.84 ps. CSI exhibits three GSB peaks at λ_det_ = 666, 794, and 814 nm and a Stark lineshape near λ_det_ = 575 nm. As discussed above, the presence of the Stark lineshape and the blue-shifted A_0_ peak are fingerprints of a CSS involving A_0_ and at least one BChl g pigment. Compared to P_800_^+^A_0_^−^, CSI exhibits a larger splitting between the two GSB peaks in the BChl Q_y_ band, suggesting the hole wavefunction in CSI may be more delocalized in this state than in P_800_^+^A_0_^−^. Given the coupling strength of ~200 cm^−1^ between Acc and P_800_^8,40^, we speculate that the hole wavefunction is delocalized over both of them in CSI. One of the most striking features in this fit is that the CSI state is an intermediate in both charge-separation pathways, whether initiated by the antenna exciton or the RC exciton. To support this claim, we compared the fits of the pump-probe under excitation at 666 and 690 nm using one to three CS pathways (Supplementary Figs. 5, 6) and the fits of the 2DES using two to three CS pathways (Supplementary Figs. 7–10). All of these fits show the presence of an intermediate CSS with the characteristics of CSI, providing strong evidence that A_0_ is the primary electron acceptor in the initial CS step, independent of the initial excitation. The intermediate CSI relaxes to the final CSS (CSF) with a time constant of 19 ps. CSF exhibits a derivative lineshape at 575 nm, a blue-shifted A_0_ peak at 666 nm and two P_800_^+^ peaks at 794 and 807 nm, as described earlier, leading to its assignment as P_800_^+^A_0_^−^.

We considered several models for the antenna relaxation, finding that a minimum of two distinct antenna excitons, A1 and A2, were needed. While A1 and A2 can be generated via direct photoexcitation of the vibronic shoulder of the BChl Q_y_ transitions, a small fraction of RC1 is also transferred to the antenna, populating A1 and subsequently A2. The spectroscopic characteristics of A1 and A2 are consistent with their assignment as antenna excitons: both lack amplitude at 668 nm indicative of A_0_ excitation. A1 exhibits GSB signals at λ_det_ = 570–590 nm and 790–810 nm and transitions to the CSS CSI with a time constant of 2.2 ps. Due to the transfer of RC1 to A1, we attribute A1 to an antenna exciton involving pigments near to the RC. In contrast, A2 exhibits GSB signals that are red-shifted relative to A1 and undergoes relatively slow backwards energy transfer to A1, leading us to assign A2 to low-energy antenna exciton(s)^14,16^. We note that, consistent with our initial proposed CS mechanism that was based on the LDMs and prior work^9–19^, a relaxed antenna exciton and/or an RC exciton involving P_800_ and/or Acc only may be present between A1 and CSI and act as the reactant for the initial CS step. However, this component cannot be isolated from A1, likely due to their similar spectral profiles and fast exchange rate.

## Discussion

The energy transfer and CS processes of the HbRC have been studied since the 1980s^9–16,18,19,41,42^. Lin and coworkers^9^ reported that excitonic equilibrium within the antenna is complete within ~0.8 ps, while CS takes place at ~25 ps at room temperature. They further claimed that energy transfer is the rate-limiting step and reported the upper limit of the intrinsic CS to be 1 ps^−1^_._ At cryogenic temperatures, Chiou et al.^16^ reported that all excitations are funneled into the lowest energy exciton prior to CS, which takes place within 55–70 ps. However, the Vos^18^ and Amesz groups^14,15^ observed fast CS occurring on the timescale of a few picoseconds upon photoexcitation of both antenna and A_0_, suggesting that excitonic equilibrium need not be established prior to fast CS.

Another debate concerns the difference in CS mechanisms upon excitation of the RC and antenna domains^14–16^. Initially, a one-step CS mechanism (i.e., hole transfer from A_0_ to P_800_ or electron transfer from P_800_ to A_0_) was used to describe CS for both RC and antenna excitation^14,15,18,41^. However, the one-step mechanism cannot explain the multiple time scales observed for CS, or “uphill” CS from the lowest energy antenna exciton^14,16,19^. To resolve these issues, Chiou et al.^16^ proposed a two-step mechanism in which A_0_ is the primary acceptor. Without knowledge of the location of A_0_ and its coupling to other pigments, the feasibility of this mechanism, and the relationship to CS mechanisms in other RCs, were unclear.

Upon resolution of the HbRC structure, Gisriel et al.^6^ proposed two different two-step CS mechanisms for the HbRC. In the first, Acc acts as the primary electron acceptor, similar to the dominant mechanism proposed for the purple bacterial RC^35,43^. In the second, Acc acts as the primary electron donor, with A_0_ playing the role of primary electron acceptor in analogy to PSI^1^ and the dominant mechanism in both PSI^33,44–46^ and PSII^33,46,47^. The LDA and global-target analysis of our 2DES data strongly favor the latter mechanism. Regardless of initial excitation, RC and antenna excitons both transition to a common intermediate CSS (CSI), in which A_0_ is the primary electron acceptor and the hole wavefunction is delocalized over Acc and P_800_. We cannot entirely rule out additional CS intermediates obscured by spectral congestion in the BChl Q_y_ region and overlapping time scales of energy equilibration and primary CS. However, our extensive consideration of other target models consistently found that any intermediates exhibiting the hallmark of a CSS also involved A_0_.

The mechanism we have proposed makes good chemical and biological sense. We have argued that the ancestral homodimeric RC used quinones as terminal electron acceptors to couple light-driven electron transfer to proton pumping across the membrane, driving ATP production. Inherent inefficiencies of quinone reduction in this RC drove the evolution of type I and type II RCs^7^. While type II RCs became heterodimeric and specialized the two branches, type I RCs added the F_X_ cluster to catalyze semiquinone dismutation; the subsequent switch to reducing ferredoxin necessitated a more reducing cluster as well as an upstream donor (A_0_) with much lower reduction potential. This explains why all type I RCs use a version of Chl a in the A_0_ site due to the considerably lower reduction potentials of chlorins, including A_0_^10^, compared to bacteriochlorins^48,49^. However, the use of Chl a as A_0_ places serious constraints on the CS mechanism (see Supplementary Fig. 14). It would be very difficult for Acc^-^ (BChl) to act as an effective electron donor to A_0_ (Chl), rendering the “purple bacterial RC model” unlikely. This means that the excited state of BChl g, which has a significantly lower potential than the ground state, must be used as the primary donor to A_0_. Our data are most consistent with a model in which the primary donor is actually an excited state delocalized across several BChl g (P_800_ and Acc). This mechanism may protect the HbRC from wasting energy through charge recombination reactions: delocalization of the hole moves cation density away from A_0_^−^ in the initial CSS, which should serve to slow charge recombination of the initial radical ion pair. This may be especially important for anoxygenic type I RCs, in which the redox gradient from BChl to Chl would be uphill if not for the use of the excited state. In contrast, the redox gradient from (B)Chl to (B)Pheo is downhill in type II RCs, as a (B)Chl is always more reducing than the corresponding (B)Pheo^48^. The subsequent step of localizing the hole to the P_800_ BChl dimer would be a natural consequence of its higher reduction potential, enabling the special pair to stabilize the hole and place it near the external side for reduction by the external donor (cytochrome c).

In conclusion, multispectral 2DES has provided insight into the excitonic structure and CS mechanism of the HbRC. Independent of whether the initial excitation occurred in the RC or antenna domain, our extensive kinetic analysis revealed A_0_ as the primary electron acceptor, with an intermediate CSS delocalized over P_800_, Acc, and A_0_. The use of delocalization across the special pair and accessory pigments could well be a general strategy used by other photochemical RCs^50^. The HbRC presents an especially tractable case, due to the chemically different pigment in the A_0_ site. Our study highlights the different ways in which nature has made use of the basic RC architecture that emerged over 3 billion years ago.

## Methods

### Sample preparation

The isolation of the HbRC is described in previous studies^11^. In brief, cells grown to late-exponential phase were harvested at 10,000 × *g* and resuspended in 50 mM MOPS buffer (pH 7). Whole cells were lysed by sonication, and membranes were pelleted by centrifugation at 200,000 × *g*. Membranes were solubilized with 1% *n*-dodecyl-β-d-maltopyranoside for 1 h, and intact membranes were removed by centrifugation at 200,000 × *g*. Solubilized membranes were passed over a diethylaminoethyl cellulose ion-exchange column equilibrated in 50 mM MOPS (pH 7.0). The flow-through, which contained the HbRC cores was collected and loaded onto a carboxymethyl-Sepharose column (CM100) equilibrated in 50 mM MOPS (pH 7.0). A brown band containing the purified HbRCs was collected in 50 mM MOPS, 20 mM MgSO_4_ buffer. Samples were concentrated with a stirred ultrafiltration cell over a 100-kDa cutoff membrane. The entire process was performed under dim green light in an anaerobic chamber. The HbRC sample was stored in a buffer solution that contains 5.38 μM HbRC, 100 mM glycine buffer (pH = 10), 0.02% *n*-dodecyl-β-d-maltoside. Before ultrafast spectroscopic measurements, the HbRC solution was treated with 20 mM dithionite to reduce the terminal [4Fe-4S] cluster (F_x_). This blocks forward electron transfer from A_0_ to F_x_, resulting in charge recombination of the P_800_^+^A_0_^−^ state in ~15 ns^10^. The 1:1 mixture of the HbRC solution and glycerol was loaded into a sealed quartz sample cell with an optical path length of 380 μm. The OD values at 786 nm and at 668 nm of the samples were 1.1 and 0.13, respectively. The transient measurements were performed at 77 K using a liquid nitrogen cryostat from Oxford Instruments.

### Spectroscopic measurements

2DES spectra were measured by using a pump-probe geometry 2DES setup (see Supplementary Note 5 for further details)^27^. The pump pulse was compressed to 12 fs. A two-phase cycling scheme was used to remove scattering and background signals. A shutter added in the probe arm removed residual scattering from the pump. In the experiments, the pulse energy of pump pulses was ~40 nJ and the beam waists (1/e^2^) for both pump and probe were ~200 μm. 2D experiments were performed under the magic-angle condition and at least three times to ensure reproducibility. The data was analyzed using home-written MatLab scripts. The chirp of the probe pulse was corrected using a third-order polynomial function as described previously^51^. The LDA was performed using the OPTIMUS software^32^. The global-target analysis was implemented using CarpetView3D (Light Conversion). Fluence dependence studies were also conducted to avoid the exciton–exciton, exciton–charge, and charge–charge annihilation (Supplementary Fig. 12).

## Supplementary information

Supplementary Information

## Data Availability

The data that support the findings of this study are available from the corresponding author upon reasonable request.

## Materials availability

The Ice1 strain of *Heliobacterium modesticaldum* is available from K.E.R. upon request.
