# Supplementary material for: Excitonic structure and charge separation in the heliobacterial reaction center probed by multispectral multidimensional spectroscopy
Source: Nat Commun. 2021 May 14;12:2801. doi: 10.1038/s41467-021-23060-9 (PMC8121816; doi:10.1038/s41467-021-23060-9)
Supplement: Supplementary file 1 — Supplementary Information [file 41467_2021_23060_MOESM1_ESM.pdf]

Supplementary Information

Excitonic structure and charge separation in the Heliobacterial Reaction Center probed by multispectral multidimensional spectroscopy

Yin Song[1], Riley Sechrist[1], Hoang H. Nguyen[1], William Johnson[2], Darius Abramavicius[3], Kevin E. Redding[2,4], and Jennifer P. Ogilvie[1]

[1]*Department of Physics, University of Michigan, Ann Arbor, MI, 48109, US*

[2]*School of Molecular Sciences, Arizona State University, Tempe, AZ, 85287, USA,*

[3]*Department of Theoretical Physics, Faculty of Physics, Vilnius University, Sauletekio 9-III, 10222 Vilnius, Lithuania*

[4]*Center for Bioenergy and Photosynthesis, Arizona State University, Tempe, AZ, 85287, USA*

Table of Contents

Supplementary Note 1 The linear absorption, pump and probe spectra. 2
Supplementary Note 2 Comparison of the photoexcited dynamics upon visible and near-IR excitation. 2
Supplementary Note 3 Lifetime density analysis of transient data upon excitation at 666 nm and 690 nm 3
Supplementary Note 4 Additional global target analysis 6
  Supplementary Note 4.1 Global target analysis of transient data upon excitation at 666 nm 6
  Supplementary Note 4.2 Global-target analysis of transient data upon excitation at 690 nm using two kinetic models 8
  Supplementary Note 4.3 Other kinetic models to fit the 2D data 10
Supplementary Note 5 Spectroscopic Measurements 15
Supplementary Note 6 Simulations 18
Supplementary Note 7 Simulations of the Stark Shift of A_0 20

Supplementary Note 1 The linear absorption, pump and probe spectra.

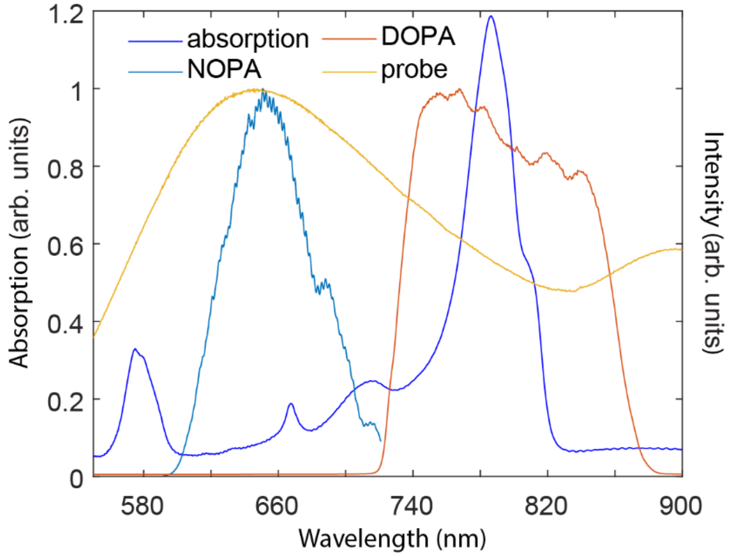

Supplementary Fig. 1 | Linear absorption spectrum (arbitrary unit = a.u.) of HbRC at 77K and the pump (i.e., non-collinear optical parametric amplifier and degenerate optical parametric amplifier) and probe laser spectra used in the 2DES measurements.

Supplementary Note 2 Comparison of the photoexcited dynamics upon visible and near-IR excitation.

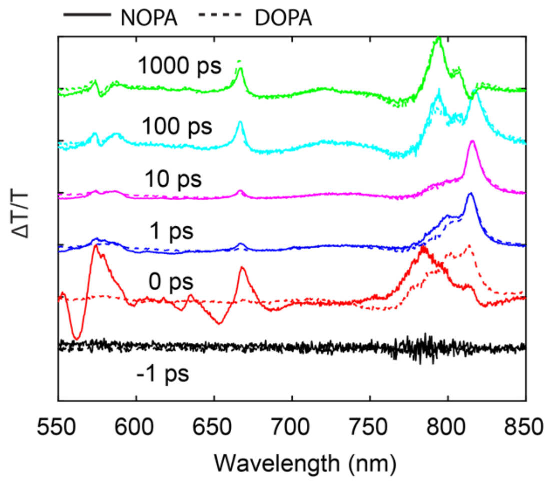

Supplementary Fig. 2 | Comparison of photoexcited dynamics under the visible NOPA pump and the near-IR pump from a degenerate OPA spanning 720-860 nm. The similarity of the two sets of transient absorption spectra at 10 ps, 100 ps and 1000 ps suggests that both conditions lead to the same charge separation pathways after 10 ps.

Supplementary Note 3 Lifetime density analysis of transient data upon excitation at 666 nm and 690 nm

Supplementary Fig. 3 displays the lifetime density maps (LDMs) with λ_{ex} = 666 nm to reveal the kinetics following RC excitation. The LDMs exhibit multiple lifetime bands with strong detection wavelength dependence. The temporal widths of the lifetime bands are determined by the uncertainty of lifetimes and the system's inhomogeneity. With excitation at 666 nm, where the primary photoexcitation is of the highest energy RC excitons, the LDMs (**Supplementary Fig. 3 panels a2, a3**) exhibit a positive-amplitude peak at τ = 0.16 ps and λ_{det} = 668 nm (i.e., decay of the GSB and SE) and a negative-amplitude peak (i.e., GSB growth) at τ = 0.10 ps and λ_{det} = 796 nm (i.e., BChl *g* Q_y bands). This component can be attributed to excitonic equilibration or vibrational relaxation within the RC. The difference between these time constants (0.10 *vs.* 0.16 ps) is likely due to overlap of the oppositely-signed components at τ = 0.10 and 0.86 ps and λ_{det} = 796 nm (**Supplementary Fig. 3 panel a3**). Subsequent energy transfers from the RC to the antenna and within the antenna are revealed by the positive-amplitude BChl Q_y peaks from 770 to 810 nm and negative-amplitude BChl Q_y peaks from 810 to 830 nm, which appear on timescales ranging from 0.70 to 1.1 ps.

The most interesting features in the LDM are the Stark lineshapes of the BChl Q_x peak (shown in **Supplementary Fig. 3 panels a1** and **b1**) at 1.7 ps and 230 ps. This observation reveals that there are two time windows for CS. The spectrum at 230 ps exhibits three negative-amplitude peaks at 666 nm, 794 nm and 807 nm, providing firm evidence for the formation of the CSS ($P_{800}^{+}A_0^{-}$). A slow CS component (τ > 100 ps) has been observed previously, although only at cryogenic temperatures[1]. We also observe the slow component following antenna excitation at 690 nm (**Supplementary Fig. 4 panels b1-b3**), suggesting a role for antenna excitons in this process.

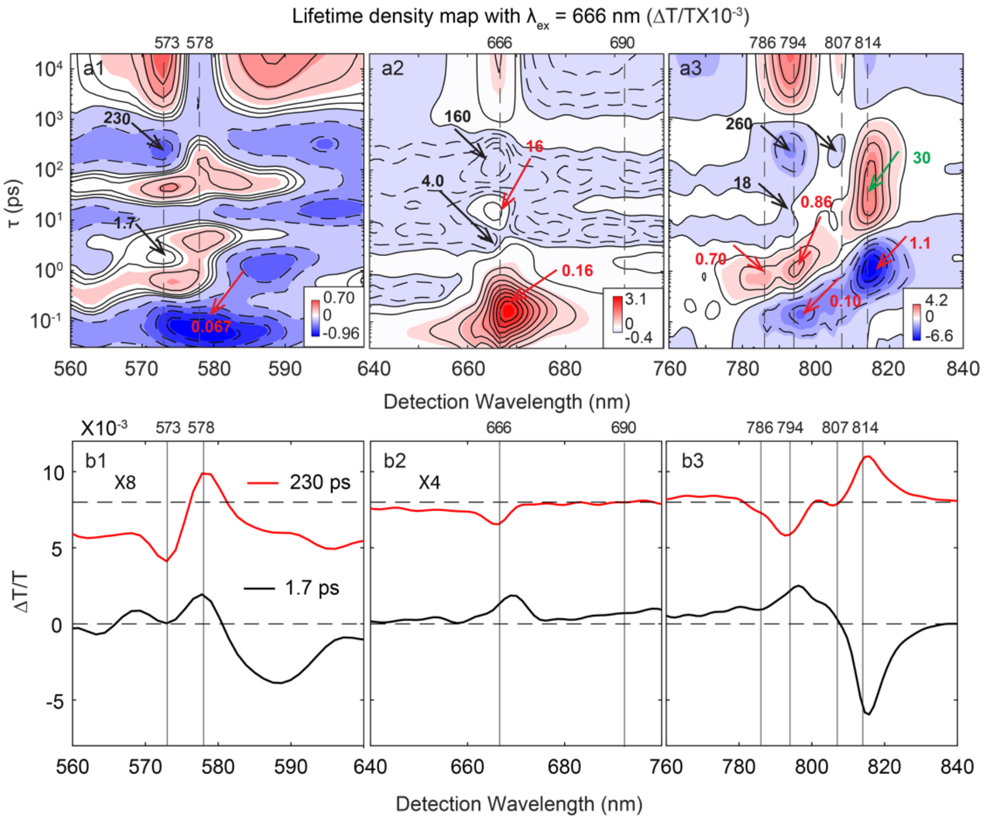

Supplementary Fig. 3 | Lifetime density analysis shows energy transfer and charge separation dynamics after excitation of the RC. **Panels a1-a3)** Lifetime density maps under excitation at 666 nm. Dashed lines indicate the various peaks of key participants during charge separation. From left to right, these dashed lines are located at 573 nm, 578 nm, 666 nm, 690 nm, 786 nm, 794 nm, 807 nm and 814 nm to enable comparison of peak locations. The arrows are used as guidance for the lifetime peaks corresponding to the crucial photoexcited dynamics as discussed in the main text. The lifetime constants are annotated beside the arrows. The arrows are color-coded for assignment of features associated with vibrational relaxation or energy transfer (red), charge separation (black), and a combination of both (green). The contour levels are -1:0.1:1 of the maximum amplitude. **Panels b1-b3)** Slice spectra at λ_{ex} = 666 nm and τ = 1.74 ps and 228 ps corresponding to the main charge separation processes. The derivative features in the BChl *g* Q_x band with minima and maxima at 573 and 578 nm reveal the formation of the charge separated states. The peaks at 666 nm indicate involvement of A_0 in these processes.

While the spectrum at 1.7 ps shows Stark lineshapes of the BChl *g* Q_x peak (**Supplementary Fig. 3 panels b1-b3**), it does not yet exhibit the other clear signatures of $P_{800}^+A_0^-$ (i.e., peaks at 666 nm, 794 nm and 807 nm), suggesting that an intermediate CSS exists on this timescale. The decay of the GSB of A_0 on this timescale is consistent with the participation of A_0 in the intermediate CSS and suggests that it may be the primary electron acceptor.

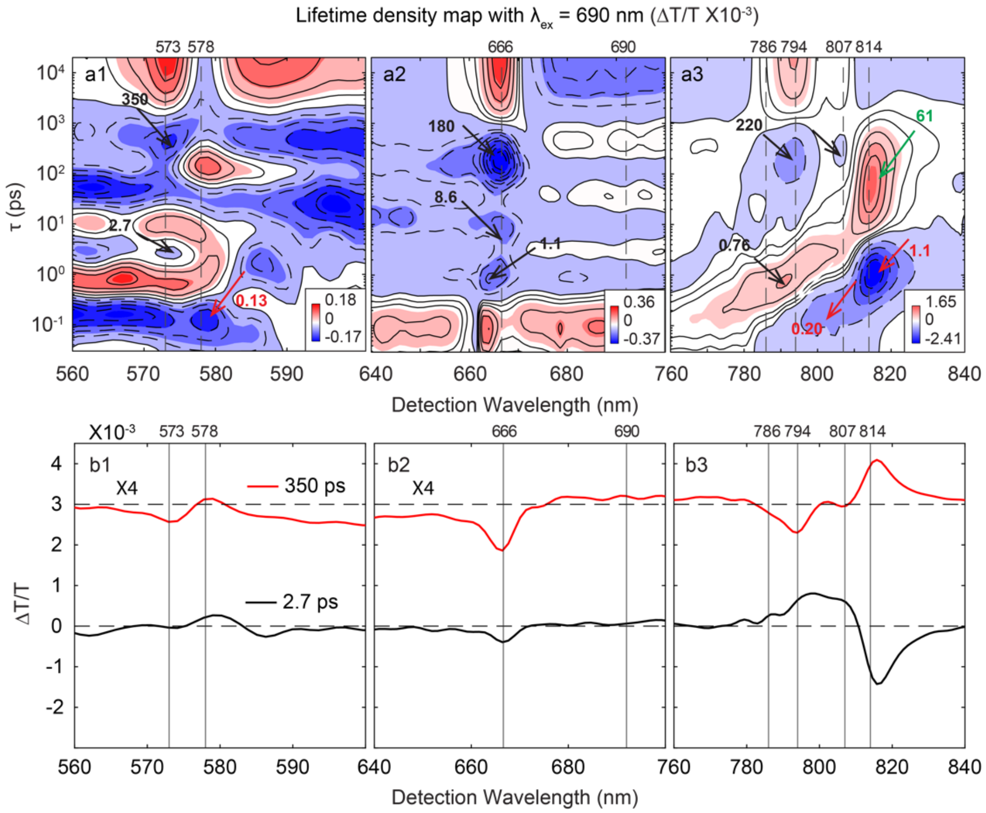

Supplementary Fig. 4 | Lifetime density analysis showing energy transfer and charge separation dynamics after antenna excitation. **Panels a1-a3)** Lifetime density maps under excitation at 690 nm. Dashed lines indicate the various peaks of key participants during charge separation. From left to right, these dashed lines are located at 573 nm, 578 nm, 666 nm, 690 nm, 786 nm, 794 nm, 807 nm and 814 nm and are also shown in the 2D spectra of Figure 2 to enable comparison of peak locations. The arrows are used as guidance for the lifetime peaks corresponding to charge separation (black), energy transfer (or vibrational relaxation, red), or a combination of both (green). The lifetime constants are annotated beside the arrows. The contour levels are -1:0.1:1 of the maximum amplitude. **Panels b1-b3)** Slice spectra at λ_{ex} = 690 nm and τ = 2.74 ps and 350 ps corresponding to the main charge separation processes. The derivative features in the BChl *g* Q_x band reveal the formation of the CSSs. The peaks at 666 nm indicate possible involvement of A_0 in this process.

Supplementary Fig. 4 displays the lifetime density analysis upon excitation at 690 nm, which primarily generates antenna excitons. The lifetime density maps (LDMs, **Supplementary Figure 4 a2, a3**) exhibit a positive-amplitude peak at τ = 0.030-0.30 ps and λ_{det} = 690-780 nm (i.e., the decay of the GSB and stimulated emission) and a negative-amplitude peak (i.e., the GSB growth) at τ = 0.20 ps, and λ_{det} = 800 nm (i.e., the BChl *g* Q_y bands). This component can be attributed to the excitonic equilibrium or vibrational relaxation within the antenna. Subsequent energy transfer between antenna excitons is revealed by the

positive-amplitude peak at τ = 0.76 ps and λ_{det} = 792 nm and a negative-amplitude band at τ = 1.1 ps and λ_{det} = 816 nm. Since energy transfer from BChl g to A_0 is not energetically favorable, three negative-amplitude A_0 peaks at τ = 1.1, 8.6 and 180 ps are attributed to charge separation. As discussed in the manuscript, the slowest charge-separation component is accompanied by a Stark line shape near 576 nm (**Supplementary Fig. 4 panel a1**) and two negative peaks at 794 and 807 nm (**Supplementary Fig. 4 panel a3**), arising from the lowest energy antenna exciton. The fastest process exhibits a Stark line shape nearby 576 nm but lacks the negative peaks at 794 and 807 nm, suggesting that the initial charge separation gives rise to an intermediate CSS on this time scale. Neither the Stark lineshape near 576 nm nor the negative peaks at 794 and 807 nm are observed at 8.6 ps. We conjecture that these features might be masked by the strong signals arising from energy transfer. Given the analysis above, we propose three possible pathways for antenna excitons: 1) Antenna exciton nearby RC → (RC)* → Intermediate CSS → $P_{800}^+A_0$; 2) Antenna exciton away from RC → Antenna exciton nearby RC → (RC)* → Intermediate CSS → $P_{800}^+A_0^-$; 3) Antenna exciton → Low-energy antenna exciton →Antenna exciton nearby RC → (RC)* → Intermediate CSS → $P_{800}^+A_0^-$. In the global-target analysis, we were not able to separate this (RC)* and the antenna exciton nearby RC probably due to their similar spectral profiles and fast exchange rates. Thus, we ignore this step in the discussion below.

Supplementary Note 4 Additional global target analysis

Supplementary Note 4.1 Global target analysis of transient data upon excitation at 666 nm

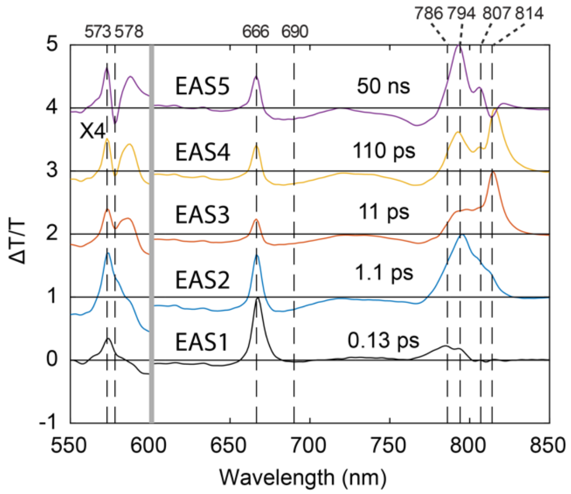

Supplementary Fig. 5 | Global-target analysis of the transient data upon excitation at 666 nm using a sequential model. The plot shows the evolution-associated spectra with the corresponding time constants. The signal in the spectral range from 550 to 600 nm is multiplied by a factor of 4 to better visualize the Stark line shape.

We used a sequential model to fit the transient data produced by excitation at 666 nm (**Supplementary Fig. 5**). Five compartments are required to obtain a satisfactory fit. The first evolution-associated spectra (EAS1) is assigned to the primary product of photoexcitation and exhibits an A_0 peak at 668 nm, a weak BChl *g* Q_x peak at 573 nm and a BChl *g* Q_y peak at 780 nm. This observation suggests that the primary photoexcitation is delocalized over A_0 and Acc, which is consistent with the excitonic assignments proposed in the manuscript. EAS1 evolves to EAS2 with a time constant of 1.1 ps via vibrational relaxation, excitonic relaxation or energy transfer. Thus, EAS2 could be a mixture of both the relaxed RC exciton and the antenna exciton. The EAS3 exhibits a Stark lineshape at 576 nm, an A_0 peak at 666 nm and a BChl *g* Q_y at 814 nm. The rapid formation of EAS3 suggests that the initial charge separation occurs at about 1.1 ps. The simultaneous appearance of the Stark lineshapes and the 814-nm feature also implies that the latter stems from a charge separated state (CSS). The spectral profile of EAS4 resembles EAS5 except that it exhibits a strong peak at 816 nm. The 816 nm peak can be attributed to the lowest energy exciton, generated either by energy transfer from the RC exciton or the antenna exciton, or by directly photoexciting the vibrational shoulders of the lowest energy excitons. The spectral evolution from EAS3 to EAS4 reveals the relaxation of the intermediate CSS to the final CSS, $P_{800}^{+}A_0^{-}$. The lowest energy exciton at 816 nm transitions to the CSS with a time constant of 110 ps, which is consistent with previous studies[2].

Supplementary Note 4.2 Global-target analysis of transient data upon excitation at 690 nm using two kinetic models

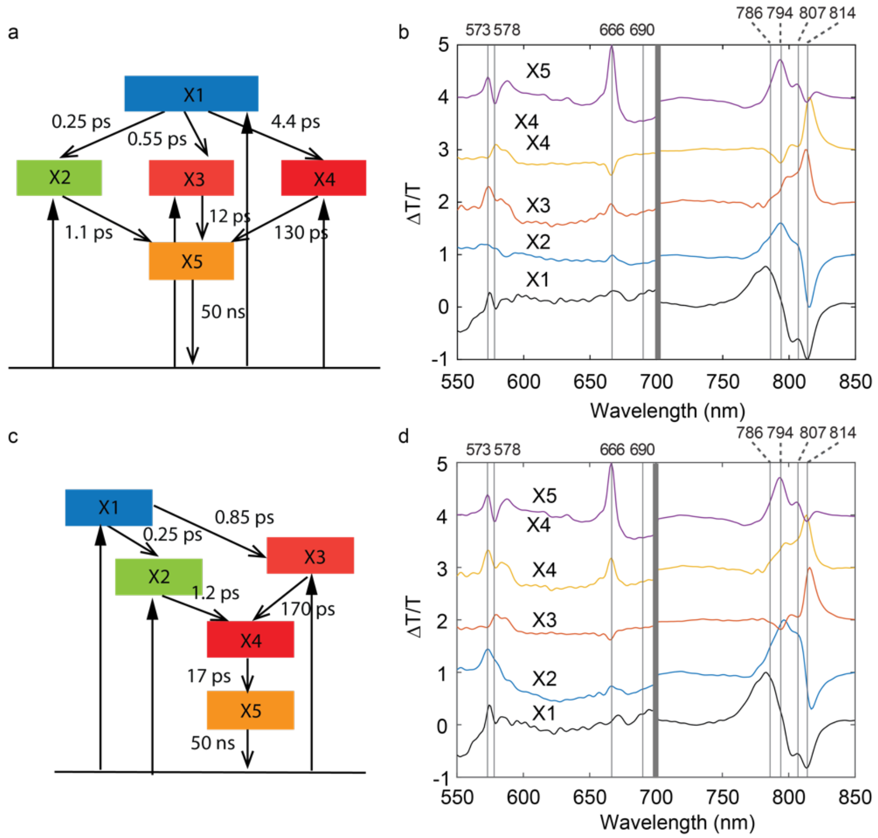

Supplementary Fig. 6 | Target analysis upon excitation at 690 nm when the antenna excitons are primary photoexcitation products. Since LDA shows that charge separation takes place on several time scales, we used kinetic models with three (a) and two (c) charge separation pathways to fit the data. In both fits, there appears a SAS (X3 in **panel b** and X4 in **panel d**) with a Chl peak, a BChl peak at 814 nm and a Stark feature at about 580 nm. Combining this observation with the LDAs, the global analysis of the transient data upon excitation at 666 nm and the target analysis in the manuscript, suggests a common intermediate CSS originating from either the high-energy RC exciton or the low-energy antenna excitons.

To test alternative charge separation models and determine whether charge separation produces a common intermediate CSS from either the antenna excitons or the high-energy RC exciton, we extracted 'pump-probe' spectra upon excitation at 690 nm from 2DES and then fit the transient data using different kinetic models. Since only antenna excitons are photoexcited in this case, if the Acc acts as the primary acceptor, these fits promise to capture the predicted intermediate state, $P_{800}^{+}Acc^{-}$.

As discussed above, LDMs (**Supplementary Fig. S4**) show charge separation signatures at 1.1 ps, 8.6, and 180 ps. Thus, we first fit the data using a kinetic model with three parallel charge separation pathways

(**Supplementary Fig. 6a**). In this model, we also take into account excitonic relaxation and vibrational relaxation, which take place from a few hundreds of femtosecond to several picoseconds, as reported in previous studies[1-4]. We find that the SAS X3 exhibits a Stark lineshape of the BChl *g* Q_x peak and an A_0 peak. The resemblance of X3 and $(P_{800}Acc)^+A_0^-$ suggests that A_0 is the primary acceptor in the initial CS step. No clear spectral signatures of a CSS are seen in X2. One feature that cannot be well explained in X4 is the significant negative-going Chl peak. We conclude that this model cannot give a satisfactory fit with physically meaningful SAS.

As shown in **Supplementary Fig. 6c**, we also considered a kinetic model involving two single step parallel pathways to reach an intermediate state (X4). The two branches leading to X4 in this model are similar to the pathways for antenna excitons in the manuscript. While these relaxation steps can be successfully included in the fit of the transient data upon a single-wavelength excitation (shown in **Supplementary Fig. 6d**) the fit of the whole 2D dataset fails to converge, likely due to the similar time scales of relaxation of the RC and the antenna excitons. Interestingly, **Supplementary Fig. 6d** shows that in the case of antenna excitation, the intermediate state X4 exhibits a Stark lineshape of the BChl *g* Q_x peak, a Chl Q_y peak at 666 nm and a BChl *g* Q_y peak at 814 nm, similar to the intermediate CSS obtained from the fits of the transient data upon excitation at 666 nm (i.e., the 11-ps component in **Supplementary Fig. 5**) and the fits of the 2D dataset in the manuscript (i.e., CSI in the **Figure 3** of the manuscript). This observation provides further strong evidence that a common CSS is involved in the charge-separation pathways and that A_0 is the primary electron acceptor, for both the antenna exciton and the high energy RC exciton.

Supplementary Note 4.3 Other kinetic models to fit the 2D data

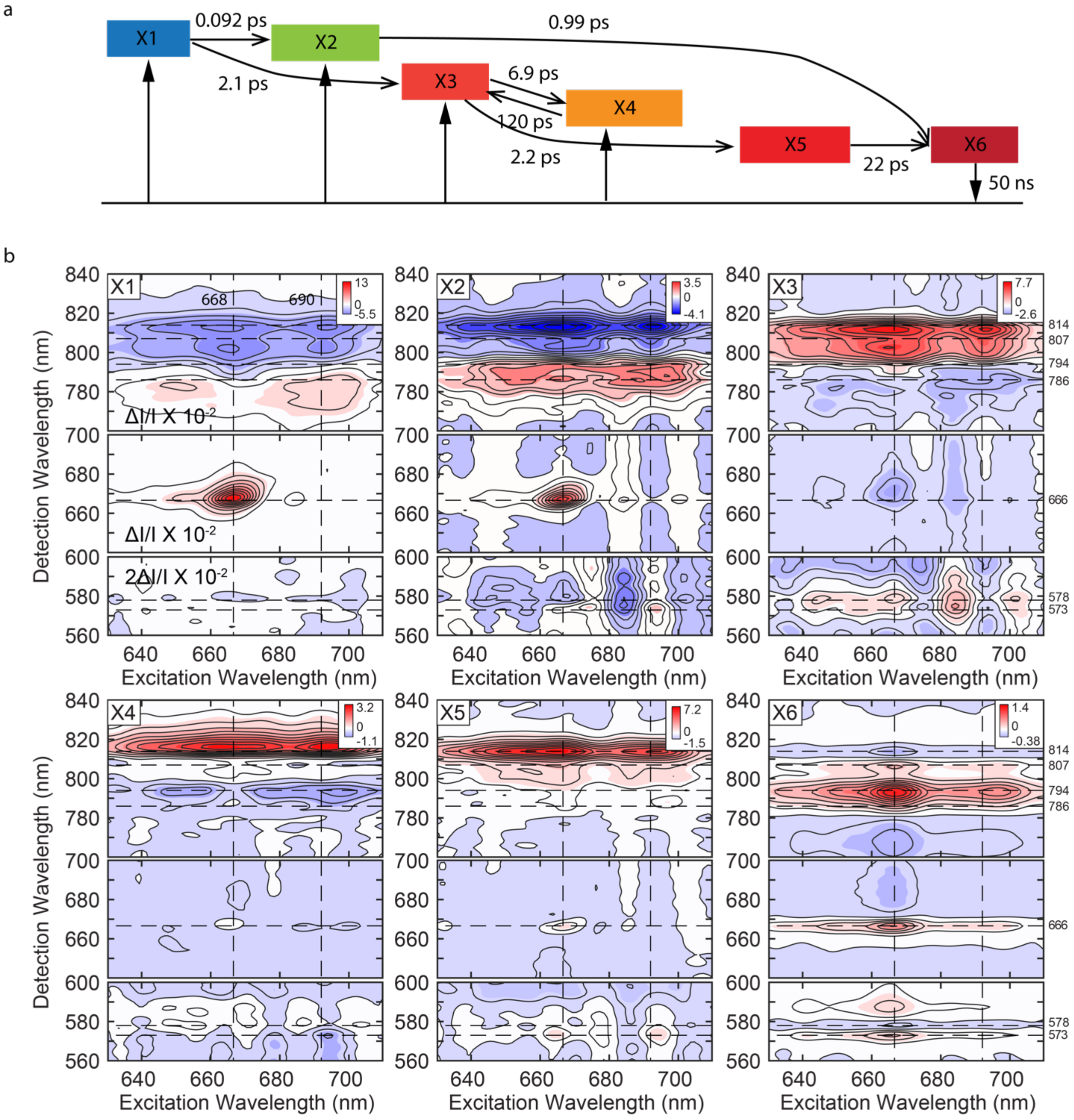

Supplementary Fig. 7 | Global-target analysis using an alternative kinetic model that includes two independent charge separation pathways for the antenna exciton and the high energy RC exciton (AccA$_0$). a) Kinetic model with time constants obtained from the fits. b) Species-associated spectra. The contour levels are -1:0.1:1 of the maximum amplitude.

We also considered other kinetic models with independent charge separation pathways for excitation of the antenna and RC domains. These kinetic models promise to capture the intermediate state of $P_{800}^{+}Acc^{-}$ if Acc acts as the primary acceptor in the charge separation pathway for the antenna exciton. Previous studies[1,2,5,6] and the LDA in the current work have shown that the ($AccA_0$) exciton transition to the CSS on the time scale of several picoseconds while for the antenna excitons, charge separation takes place in three time windows ranging from several picoseconds to hundreds of picoseconds. **Supplementary Fig. 7** shows target analysis using a kinetic model with two charge separation pathway: 1) one for the ($AccA_0$) exciton and 2) one for the antenna excitons. Since charge separation takes place in three time windows, we included a back energy transfer pathway from the lowest energy exciton to the antenna exciton, which takes into account of the slowest charge separation on the time scale of hundreds of picosecond. However, two features in SASs indicates that this model does not work well. The peaks in the SASs, X2 and X3 are centered at the same wavelengths but their amplitudes have opposite signs. This is an artifact which is often observed in the exponential fit. In the charge-separation pathway for antenna excitons, the SASs, X4 and X5 exhibits weak Chl *a* peaks at 666 nm, suggesting that A_0 may be involved in the initial step(s) of charge separation.

The analysis above also shows that for the antenna-exciton pathway (X3→X5→X6), the final charge separation step takes place with a time constant of 22 ps. Previous studies[2,7] have proposed that the rate-limiting step for this pathway may be the energy-transfer step from the antenna to the RC and charge separation may take place at about 1 ps. Therefore, we fit the 2D data using the same kinetic model but put a constraint of the charge separation rate (X5→X6) to be in the range of $(5\ ps)^{-1}$ to $(0.5\ ps)^{-1}$. The fitting results are shown in **Supplementary Fig. 8**. We find that SAS X5 exhibits a Chl *a* peak at 666 nm and a Stark shift feature at the Q_x band of BChl *g*. This observation suggests the involvement of the A_0 Chl in the initial charge-separated step.

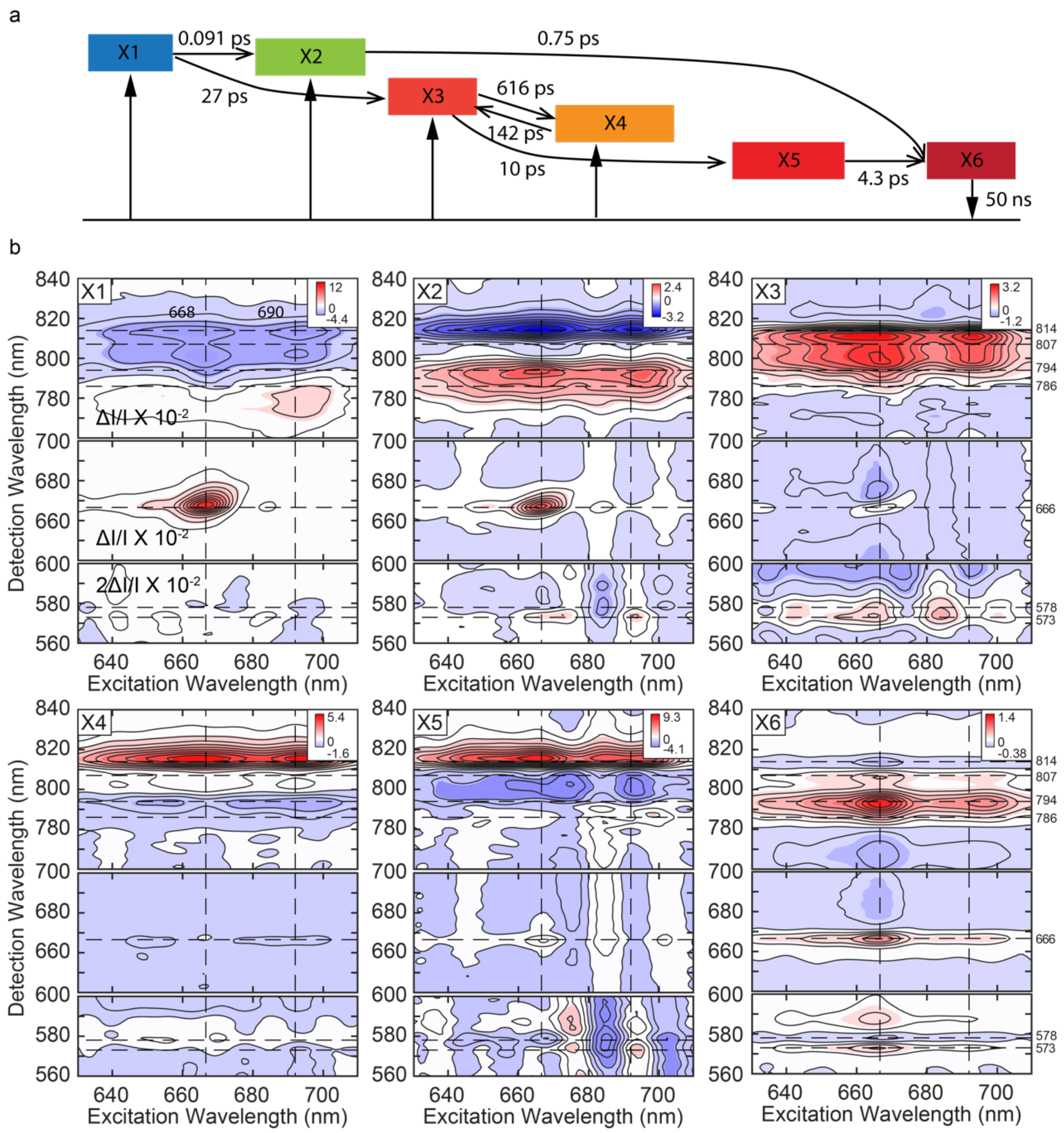

Supplementary Fig. 8 | Global-target analysis using an alternative kinetic model that includes two independent charge separation pathways for the antenna exciton and the high energy RC exciton ($AccA_0$). **a)** Kinetic model with time constants obtained from the fits. **b)** Species-associated spectra. The contour levels are -1:0.1:1 of the maximum amplitude.

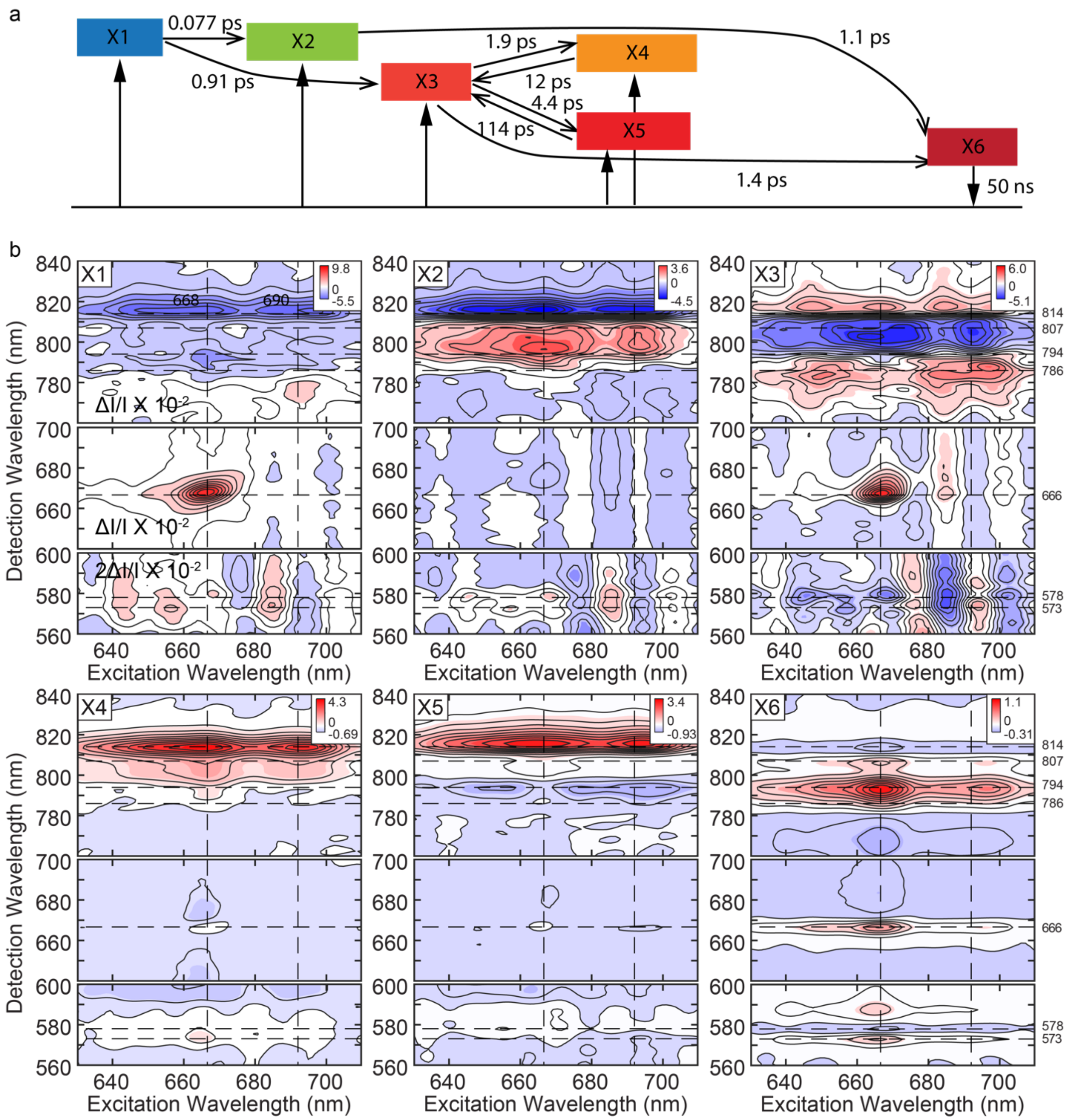

Supplementary Fig. 9 | Global-target analysis using an alternative kinetic model that includes two independent charge separation pathways for the antenna exciton and the high energy RC exciton (AccA$_0$). **a)** Kinetic model with time constants obtained from the fits. **b)** Species-associated spectra. The contour levels are -1:0.1:1 of the maximum amplitude.

Supplementary Fig. 9 displays the global fits of the 2DES data using another kinetic model with two charge separation pathways: 1) one for the (AccA$_0$) exciton and 2) one for the antenna excitons. Different from the previous kinetic models, both forward and backward energy transfer between all antenna

excitons (X4⇆X3⇆X5) are considered, and charge separation takes place via a one-step mechanism. However, this fit does not produce a satisfactory result. First, SAS of an antenna exciton X3 exhibits an A_0 peak while the RC exciton X2 does not. Second, the peaks at around 575 nm and 800 nm in the SASs, X2 and X3 are centered at the same wavelengths but their amplitudes have opposite signs, which is an artifact in the exponential fit.

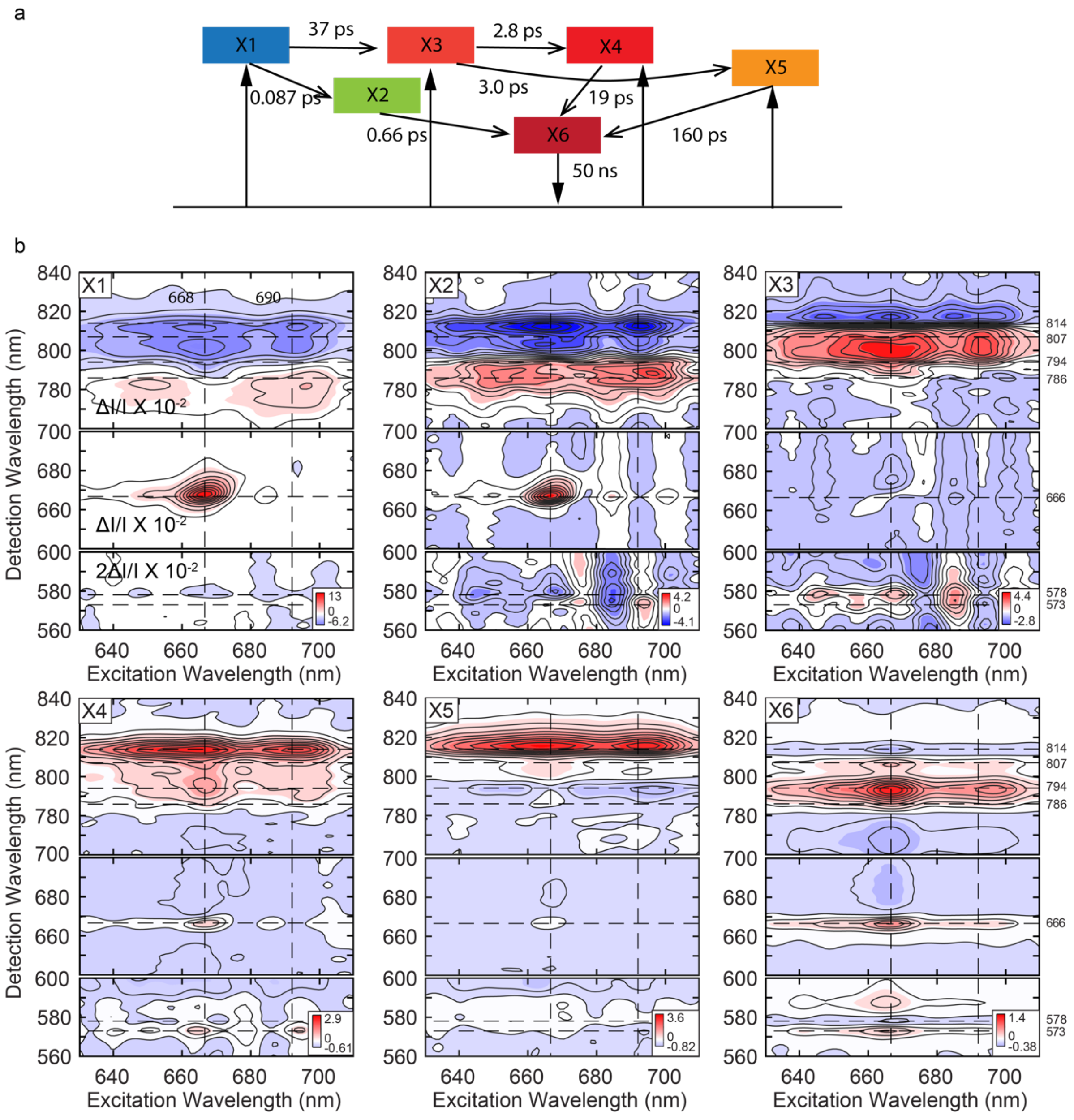

Supplementary Fig. 10. Global-target analysis using an alternative kinetic model that includes three independent charge separation pathways for the antenna exciton and the high energy RC exciton ($AccA_0$).

a) Kinetic model with time constants obtained from the fits. b) Species-associated spectra. The contour levels are -1:0.1:1 of the maximum amplitude.

Supplementary Fig. 10 displays the global fits of the 2DES data using a kinetic model with three charge separation pathways: 1) one for the (AccA$_0$) exciton and 2) two for the antenna excitons. In the fit, we find that the antenna-exciton pathway exhibits an SAS (X4) with a Stark feature in the BChl *g* Q_x band, an A_0 peak at 666 nm and two BChl *g* Q_y peaks at 790 nm and 814 nm. This feature resembles the intermediate CSS obtained from the target analysis using the kinetic model in the manuscript, as well as the global-target analysis of the transient data upon single wavelength excitation at 666 nm and 690 nm. This observation is further support that an intermediate CSS is involved in the charge separation pathway initiated by the antenna exciton, and that A_0 is the primary electron acceptor. A kinetic model of four charge-separation pathways has also been tested. However, no convergent answer was obtained for the fits using four charge separation pathways.

Supplementary Note 5 Spectroscopic Measurements

2DES spectra were measured by using a pump-probe geometry 2DES setup as described previously[8]. Briefly, two regenerative amplifiers (i.e., Spitfire Pro and Solstice from Spectra Physics) seeded by a Ti:Sapphire oscillator (MaiTai SP from Spectra Physics) are used as the laser sources. The output from the Spitfire Pro (40-fs pulses, 4-mJ, 800 nm, 500 Hz) feeds a home-built two-stage non-collinear optical parametric amplifiers (NOPAs)[9] used as the pump beam. The pump beam is sent through a pre-compensating combination of two gratings and two prisms and then into an acousto-optic pulse shaper (Dazzler, Fastlite), where a compressed pulse pair with a programmable time delay (t_1) is generated. As shown in **Supplementary Fig. 11**, the pump NOPA are compressed to 12 fs using the SPEAR method[10]. The 1-kHz output from the Solstice, chopped at 500 Hz, pumps a commercial optical parametric amplifier (TOPAS Twins from Light Conversion) to generate a 1300-nm seed beam. A portion of the 1300-nm seed beam is then focused into a 1-mm sapphire plate to generate the white light continuum (i.e., the probe

beam). The rest of the 1300-nm beam, combined with the rest of the Solstice output (i.e., 1 mJ), are used to feed a home-built degenerate optical parametric amplifier (DOPA)[11] to generate the near-IR pump beam. The pump and probe pulses are focused at the sample position to generate the third-order 2DES signal, which is detected by a CCD camera (Princeton Instruments PIXIS 100B). During the experiments, t_1 is scanned using the Dazzler from 0 to 150 fs with time steps of 2 fs. The 2DES data have been collected in a rotating frame. The pump-probe delay (T) is controlled by an optical delay line (DDS220, Thorlabs Inc.) and scanned from -5 to 1000 ps for both the pump-probe and 2DES. A two-phase cycling scheme is used as described previously to remove scattering and background signals[12]. A shutter added in the probe arm removed residual scattering from the pump. In the experiments, the pulse energy of pump pulses was ~40 nJ and the beam waists ($1/e^2$) for both pump and probe were ~200 μm. 2D experiments were performed under the magic-angle condition and at least three times to ensure reproducibility. The data is analyzed using home-written MatLab scripts. The chirp of the probe pulse has been corrected using a third-order polynomial function as described previously[13]. The lifetime density analysis is performed using the OPTIMUS software[14]. The global-target analysis is performed using CarpetView3D (Light Conversion). Fluence dependence studies are also performed to avoid the exciton-exciton, exciton-charge, and charge-charge annihilation (**Supplementary Fig. 12**).

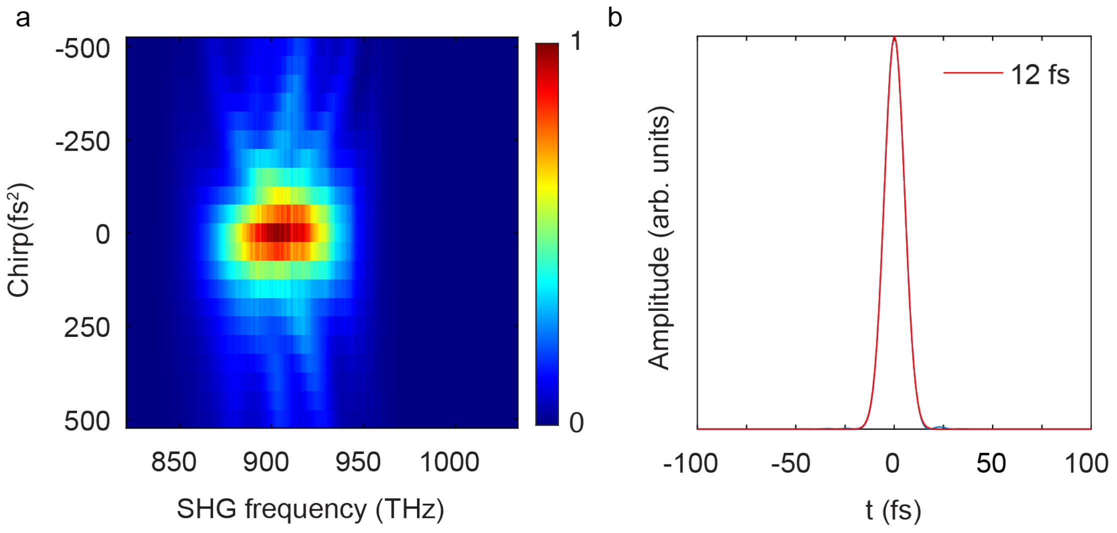

Supplementary Fig. 11 | a) the second-harmonic generation signals during a chirp scan[10] and b) the estimated pump pulse duration.

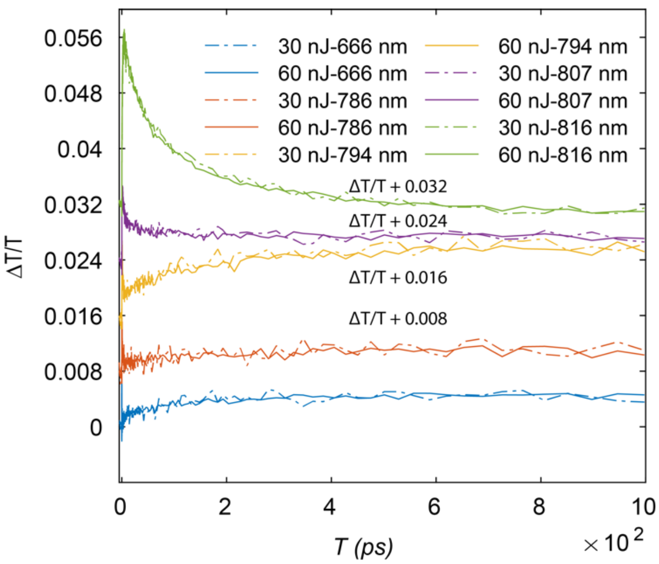

Supplementary Fig. 12 | Photoexcited dynamics at selective peaks in the HbRC under two different fluence conditions.

Supplementary Note 6 Simulations

Supplementary Table 1 | Hamiltonian with parameters extracted from the Kimura model[15]. Transition energies are on the diagonal, while intermolecular coupling, J_{ij}, is in the off diagonal region (units are cm^{-1}).

	BChl-g' 1	BChl-g 2	Chl-aF 3	BChl-g' 31	BChl-g 32	Chl-aF 33
BChl-g' 1	12739	-208	25	250	-48	3
BChl-g 2	-208	12739	307	-48	54	-15
Chl-aF 3	25	307	14925	3	-15	14
BChl-g' 31	250	-48	3	12739	-208	25
BChl-g 32	-48	54	-15	-208	12739	307
Chl-aF 33	3	-15	14	25	307	14925

Supplementary Table 2| Pigment participation probability for the excitons in the Kimura model[15]. The excitons are listed in the order of ascending energy.

	Exciton 1	Exciton 2	Exciton 3	Exciton 4	Exciton 5	Exciton 6
Position	(809.1 nm)	(795.8 nm)	(784.6 nm)	(757.9 nm)	(669.8 nm)	(671.1 nm)
Lifetime	38.7 ps	0.56 ps	0.37 ps	0.69 ps	7.9 ps	3.3 ps
BChl-g' 1	0.355	0.146	0.145	0.354	0.000	0.000
BChl-g 2	0.142	0.348	0.348	0.144	0.010	0.009
Chl-aF 3	0.003	0.006	0.007	0.003	0.490	0.491
BChl-g' 31	0.355	0.146	0.145	0.354	0.000	0.000
BChl-g 32	0.142	0.348	0.348	0.144	0.010	0.009
Chl-aF 33	0.003	0.006	0.007	0.003	0.490	0.491

Supplementary Table 3 | Hamiltonian with modified BChl-g site energies. Transition energies are on the diagonal, while intermolecular coupling, J_{ij}, is in the off diagonal region (units are cm^{-1}).

	BChl-g' 1	BChl-g 2	Chl-aF 3	BChl-g' 31	BChl-g 32	Chl-aF 33
BChl-g' 1	12739	-208	25	250	-48	3
BChl-g 2	-208	13150	307	-48	54	-15
Chl-aF 3	25	307	14925	3	-15	14
BChl-g' 31	250	-48	3	12739	-208	25
BChl-g 32	-48	54	-15	-208	13150	307
Chl-aF 33	3	-15	14	25	307	14925

Supplementary Table 4 | Pigment participation probability for the excitons in the modified BChl-g site energy model. The excitons are listed in the order of ascending energy.

	Exciton 1	Exciton 2	Exciton 3	Exciton 4	Exciton 5	Exciton 6
Position	(804.6 nm)	(780.6 nm)	(764.4 nm)	(747.5 nm)	(669.3 nm)	(670.7 nm)
Lifetime	11.6 ps	0.57 ps	0.82 ps	1.1 ps	10 ps	3.8 ps
BChl-g' 1	0.466	0.328	0.034	0.172	0.000	0.000
BChl-g 2	0.033	0.168	0.452	0.318	0.014	0.013
Chl-aF 3	0.001	0.004	0.013	0.009	0.486	0.487
BChl-g' 31	0.466	0.328	0.034	0.172	0.000	0.000
BChl-g 32	0.033	0.168	0.452	0.318	0.014	0.013
Chl-aF 33	0.001	0.004	0.013	0.009	0.486	0.487

The simulations use the Kimura and modified Kimura exciton model[15] with Hamiltonian parameters given in **Supplementary Tables 1, 3**. The single exciton states are obtained from the molecular excitation basis, where only one molecule is in its excited state while other molecules are in their ground states. In **Supplementary Tables 2, 4** pigment participation probabilities (squared wavevectors) for the exciton models are presented directly as they follow from Hamiltonians in **Supplementary Tables 1, 3** (no fluctuations are introduced). The double exciton manifold is created by assuming the bosonic relations between molecular excitations with anharmonic corrections. Each molecule can be excited twice into an overtone state with energy penalty (200 cm^{-1} anharmonicity). Creation of such state on molecule i from its single excitation additionally has a reduced transition dipole $\mu_{i,12}$ with the same dipole direction as the original molecular excitation $\mu_{i,01}$, according to $\mu_{i,12} = \sqrt{2}/2\, \mu_{i,01}$. Correspondingly, the coupling between combination state of molecules i and j and the overtone of molecule i is rescaled to value $|\mu_{i,12}|/|\mu_{i,01}| J_{ij}$ in accord with dipole-dipole coupling.

To reflect protein conformational disorder additional static fluctuations are introduced in the calculation of the spectra. Statistical ensemble averaging over uncorrelated molecular transition energy disorder is performed numerically. The site energy inhomogeneity (diagonal disorder full-width at half-maximum, i.e., FWHM of a Gaussian distribution) is 120 cm^{-1} for all sites.

Homogeneous spectral linewidth is included by assuming thermal fluctuations of molecular transition energies. The fluctuating model is realized by a modified Redfield approach, where the optical response functions are obtained within the adiabatic limit during electronic coherence periods. Diagonal system–bath fluctuations are included exactly using the cumulant expansion, truncated at the second order, and off-diagonal fluctuations are included within the Markovian second order perturbation theory (standard Redfield theory) [16,17]. The spectral density used is the same as in the Kimura model[15]. These approximations are consistent with the conditions where only zero population delay time is simulated, i. e. excited state vibrational thermalization is excluded. The off-diagonal fluctuations in the present simulations causes only lifetime broadening effects; the corresponding lifetimes are presented in **Supplementary Tables 2, 4**.

Supplementary Note 7 Simulations of the Stark Shift of A_0

To verify that the blue shift of the A_0 GSB at 946 ps is caused by the Stark effect, we simulated the Stark spectrum of A_0 in the passive branch induced by P_{800}^+ A_0^-. As described in a previous study[4], the Stark shift ($\Delta\upsilon$) can be calculated using the following formula:

$$\Delta\upsilon = -\Delta\boldsymbol{\mu}\cdot\boldsymbol{F} - \frac{1}{2}\boldsymbol{F}\cdot\Delta\boldsymbol{\alpha}\cdot\boldsymbol{F}$$

where the changes in permanent dipole moment ($\Delta\mu$) and polarizability ($\Delta\alpha$) of Chl a are set to 0.5 D/f and 1.5 $Å^3/f^2$, respectively, where f is the local field correction factor and is set to 1. The angle θ between ($\Delta\mu$) and the electric field is calculated using the HbRC crystallographic data[18]. The orientation of dipole moment is deviated from transition dipole moment by 15° according to the previous study[19]. The Stark spectrum of A_0 in the presence of $P_{800}^+A_0^-$ is shown in **Supplementary Fig. 13**. Furthermore, we also simulated the overall transient absorption spectrum in the A_0 Q_y band by summing the A_0 GSB spectrum and the Stark spectrum of A_0 in the passive branch. In this calculation, we estimated that about 6% of A_0 is photoexcited according to our transient absorption measurement. As shown in **Supplementary Fig. 13**, our simulation is consistent with the experimental observation, supporting our assignment that the blue shift of the A_0 GSB peak is due to the Stark effect.

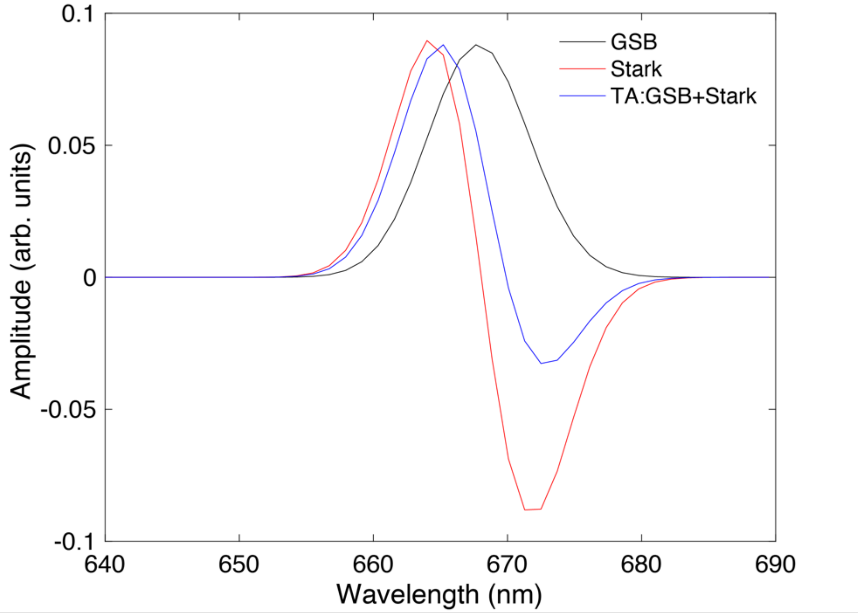

Supplementary Fig. 13 | Simulations of the electrochromic shift of A_0 (red) in the passive branch due to the presence of $P_{800}^{+}A_0^{-}$. Also shown is the ground state bleach signal (GSB) from the active branch A_0 (black). These two contributions are combined to simulate the resulting transient absorption spectrum (blue) in the A_0 Q_y region.

Supplementary Note 8 1-electron reduction potentials of cofactors in the core RC

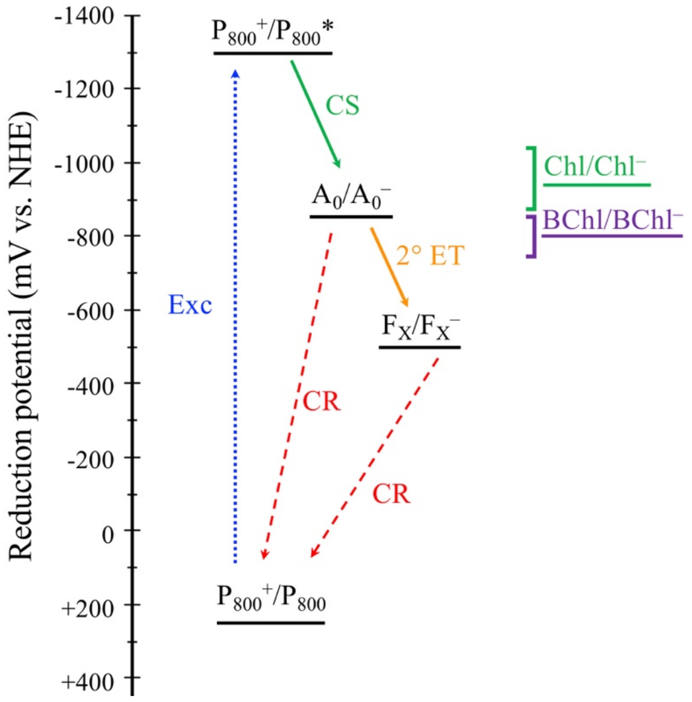

Supplementary Fig. 14 Scheme of electron transfer within the HbRC, with the 1-electron reduction potentials of cofactors plotted on an inverse potential scale (vs. the normal hydrogen electrode, NHE). The reduction potentials of P_{800} and F_X have been measured[20,21], and that of A_0 has been estimated[21] . For

reference, the reduction potentials of Chl *a* and BChl *a* determined electrochemically are indicated on the right side; the bar is the mean of the reported values, whose range is indicated by the brackets[22]. (Although the potential of BChl *g* has not been determined experimentally, due to its tendency to irreversibly oxidize, its potential was calculated to be only 10 mV more reducing than that of BChl *a*[23].) Arrows indicate various processes occurring within the RC: excitation (Exc, blue dotted), charge separation (CS, green solid), secondary electron transfer (2° ET, orange solid), and charge recombination (CR, red dashed).

Supplementary References:

1 Neerken, S., Aartsma, T. J. & Amesz, J. Pathways of Energy Transformation in Antenna Reaction Center Complexes of Heliobacillus mobilis. *Biochemistry* **39**, 3297-3303, doi:10.1021/bi992433o (2000).

2 Neerken, S. & Amesz, J. The antenna reaction center complex of heliobacteria: composition, energy conversion and electron transfer. *Bba-Bioenergetics* **1507**, 278-290, doi:Doi 10.1016/S0005-2728(01)00207-9 (2001).

3 Chiou, H.-C., Lin, S. & Blankenship, R. E. Time-Resolved Spectroscopy of Energy Transfer and Trapping upon Selective Excitation in Membranes of Heliobacillus mobilis at Low Temperature. *The Journal of Physical Chemistry B* **101**, 4136-4141, doi:10.1021/jp963384h (1997).

4 Chauvet, A. *et al.* Temporal and spectral characterization of the photosynthetic reaction center from Heliobacterium modesticaldum. *Photosynth Res* **116**, 1-9, doi:10.1007/s11120-013-9871-9 (2013).

5 Liebl, U., Lambry, J. C., Breton, J., Martin, J. L. & Vos, M. H. Spectral equilibration and primary photochemistry in Heliobacillus mobilis at cryogenic temperature. *Biochemistry* **36**, 5912-5920, doi:DOI 10.1021/bi9625197 (1997).

6 Liebl, U. *et al.* Energy and Electron Transfer upon Selective Femtosecond Excitation of Pigments in Membranes of Heliobacillus mobilis. *Biochemistry* **35**, 9925-9934, doi:10.1021/bi960462i (1996).

7 Lin, S., Chiou, H. C., Kleinherenbrink, F. A. & Blankenship, R. E. Time-resolved spectroscopy of energy and electron transfer processes in the photosynthetic bacterium Heliobacillus mobilis. *Biophysical Journal* **66**, 437-445, doi:10.1016/S0006-3495(94)80794-5 (1994).

8 Song, Y. *et al.* Multispectral multidimensional spectrometer spanning the ultraviolet to the mid-infrared. *Rev Sci Instrum* **90**, doi:10.1063/1.5055244 (2019).

9 Wilhelm, T., Piel, J. & Riedle, E. Sub-20-fs pulses tunable across the visible from a blue-pumped single-pass noncollinear parametric converter. *Opt Lett* **22**, 1494-1496, doi:Doi 10.1364/Ol.22.001494 (1997).

10 Wilcox, D. E. & Ogilvie, J. P. Comparison of pulse compression methods using only a pulse shaper. *J. Opt. Soc. Am. B* **31**, 1544-1554, doi:10.1364/JOSAB.31.001544 (2014).

11 Siddiqui, A. M., Cirmi, G., Brida, D., Kartner, F. X. & Cerullo, G. Generation of < 7 fs pulses at 800 nm from a blue-pumped optical parametric amplifier at degeneracy. *Opt Lett* **34**, 3592-3594, doi:Doi 10.1364/Ol.34.003592 (2009).

12 Fuller, F. D., Wilcox, D. E. & Ogilvie, J. P. Pulse Shaping Based Two-dimensional Electronic Spectroscopy in a Background Free Geometry. *Optics Express* **22**, 1018-1027, doi:10.1364/oe.22.001018 (2014).

13 Tekavec, P. F., Myers, J. A., Lewis, K. L. M., Fuller, F. D. & Ogilvie, J. P. Effects of chirp on two-dimensional Fourier transform electronic spectra. *Opt. Express* **18**, 11015-11024, doi:10.1364/Oe.18.011015 (2010).

14 Slavov, C., Hartmann, H. & Wachtveitl, J. Implementation and evaluation of data analysis strategies for time-resolved optical spectroscopy. *Anal Chem* **87**, 2328-2336, doi:10.1021/ac504348h (2015).

15 Kimura, A. & Itoh, S. Theoretical Model of Exciton States and Ultrafast Energy Transfer in Heliobacterial Type I Homodimeric Reaction Center. *J Phys Chem B* **122**, 11852-11859, doi:10.1021/acs.jpcb.8b08014 (2018).

16 Abramavicius, D., Palmieri, B., Voronine, D. V., Sanda, F. & Mukamel, S. Coherent Multidimensional Optical Spectroscopy of Excitons in Molecular Aggregates; Quasiparticle versus Supermolecule Perspectives. *Chemical Reviews* **109**, 2350-2408, doi:10.1021/cr800268n (2009).

17 Abramavicius, D., Valkunas, L. & Mukamel, S. Transport and correlated fluctuations in the nonlinear optical response of excitons. *Epl-Europhys Lett* **80**, doi:10.1209/0295-5075/80/17005 (2007).

18 Gisriel, C. *et al.* Structure of a symmetric photosynthetic reaction center-photosystem. *Science* **357**, 1021-1025, doi:10.1126/science.aan5611 (2017).

19 Gelzinis, A., Abramavicius, D., Ogilvie, J. P. & Valkunas, L. Spectroscopic properties of photosystem II reaction center revisited. *Journal of Chemical Physics* **147**, doi:10.1063/1.4997527 (2017).

20 Prince, R. C., Gest, H. & Blankenship, R. E. Thermodynamic Properties of the Photochemical-Reaction Center of Heliobacterium-Chlorum. *Biochim Biophys Acta* **810**, 377-384, doi:Doi 10.1016/0005-2728(85)90224-5 (1985).

21 Ferlez, B. *et al.* Thermodynamics of the Electron Acceptors in Heliobacterium modesticaldum: An Exemplar of an Early Homodimeric Type I Photosynthetic Reaction Center. *Biochemistry* **55**, 2358-2370, doi:10.1021/acs.biochem.5b01320 (2016).

22 Watanabe, T. & Kobayashi, M. in *Chlorophylls* (ed Hugo Scheer) Ch. Electrochemistry of Chlorophylls, 287-303 (CRC Press, Boca Raton, FL, 1991).

23 Fajer, J. *et al.* in *Antennas and Reaction Centers of Photosynthetic Bacteria* (ed Maria Elisabeth Michel-Beyerle) Ch. Experimental, structural and theoretical models of bacteriochlorophylls a, d, and g, 324 (Springer-Verlag, Berlin, 1985).
